# Robustness of zero-augmented models over generalized linear models in analysing fertility data in Nigeria

**DOI:** 10.1186/s13104-019-4852-5

**Published:** 2019-12-18

**Authors:** Yusuf Olushola Kareem, Imran O. Morhason-Bello, Ayo Stephen Adebowale, Joshua Odunayo Akinyemi, Oyindamola Bidemi Yusuf

**Affiliations:** 1 Department of Epidemiology and Medical Statistics, Faculty of Public Health, College of Medicine, University of Ibadan, Ibadan, Nigeria; 2 Department of Obstetrics and Gynaecology, Faculty of Clinical Sciences, College of Medicine, University of Ibadan, Ibadan, Nigeria

**Keywords:** Zero-augmented models, Fertility, Best fit model, Nigeria

## Abstract

**Objective:**

Fertility is a count data usually rightly skewed and exhibiting large number of zeros than the distributional assumption of the generalized linear models (GLMs). This study examined the robustness of zero-augmented models over GLMs to fit fertility data across regions in Nigeria. The 2013 Nigeria Demographic and Health Survey data were used. The fertility models fitted included: Poisson, negative binomial, zero-inflated Poisson, zero-inflated negative binomial, hurdle Poisson and hurdle negative binomial. Akaike Information Criteria (AIC) and Bayesian Information Criteria (BIC) were used to identify the model with best fit (α = 0.05).

**Results:**

The percentage of zero count in the fertility responses were 21.3, 23.9, 31.1, 30.7, 37.6 and 42.4 in North West, North East, North Central, South West, South South and South East regions respectively. In all the six regions in Nigeria, the zero-augmented models were better than the generalized linear models except for North Central. Extensively, the zero-augmented negative binomial based models were of better fit than their Poisson based counterparts; or in rare cases maybe indistinguishable. However, specific family of zero-augmented model is recommended for each region in Nigeria.

## Introduction

Count events frequently occur in all disciplines. In demography, count data like number of children ever born, number of deaths, and number of migration times have been previously modelled by Poisson regression [1]. One of the important assumptions guiding the use of Poisson distribution; is the equality of mean and variance which may not be feasible in reality. If this assumption is violated, the estimation method will produce biased estimates, inefficient standard errors, and misleading confidence interval and p-values [2]. Based on this limitation, researchers have recommended the use of negative binomial distribution which have an additional parameter that accounts for the usual occurrence of over-dispersion in count outcomes; thus, relaxing the constraint of equality of mean and variance [3].

Researchers have also argued that, count events are mainly characterized with large number of zeros [4–7] and this situation make modeling count data using both Poisson and negative binomial model inappropriate. Although, Poisson and negative binomial distribution assume possibilities of having zero counts but data may consist of large number of zero responses which violate the distributional assumptions of both models often referred to as the excess zero problems. Several studies have modelled fertility experience based on the distribution of the fertility pattern in different countries [3, 8–14] with a view to identifying factors influencing fertility. In Nigeria, the determinants of fertility have been examined using Poisson regression to account for the count nature of the variable [9, 11] and also negative binomial to account for over-dispersion or heterogeneity [3, 8]. Aside the limitation of the use of Poisson and negative binomial models for fertility data in Nigeria, the analysis is often conducted at national level thus neglecting some of the consequences of cultural diversities at regional level.

Nigeria has six regions defined by sociocultural differences which have implication on fertility. Striking variation exists in fertility across these regions ranging from total fertility rate (TFR) of 4.3 in South South, to 6.7 in North West [15]. Nigeria is the most populous country in Africa with population figure of about 200 million, the population of each of the six regions in the country is more than that of some countries like Togo, Republic of Benin, Liberia, Malawi, to mention a few [16]. Thus, modelling fertility data at national level and with the use of a particular model is likely to be fraught with hidden errors due to the peculiarities of the number of zeros and level of skewness inherent across regional data structures. Therefore, different models may be suitable for fertility at different regions. The current study extends [7] and modelled fertility data in each of the regions in Nigeria with six different distributions and evaluates the performance of the models for their suitability in each region.

## Main Text

### Methods

#### Data collection and utilization

The 2013 National Demography and Health Survey (NDHS) dataset was used for the implementation of the model fit. Data collection procedure involved a multi-stage cluster sampling technique. Prior to the survey, Nigeria was demarcated into smaller units regarded as enumeration areas (EAs) called clusters. This demarcation takes into consideration of the state boundaries to prevent merging of clusters within states. The respondents were selected from each cluster based on rural–urban allocation of specific numbers of clusters in the country. The current study used individual recode data with the information provided by women of childbearing age (15–49 years). Further information about the sampling strategy used for data collection can be accessed in the data originator’s website [15].

#### Data management

The outcome variable of interest was fertility which was measured by the number of children ever born (CEB), obtained from a total sample of 38,948 women. The data were weighted and the clustering effect was adjusted for in the various count models but unweighted for the skewness test and descriptive summaries of children (Additional file 1). To examine the correlation between CEB and background characteristics of women, a pairwise correlation test based on Bonferroni correction [17] for each region was conducted, 12 variables were used for the model fit: residence, women educational level, religion, ethnicity, wealth index, contraceptive use, currently residing with partner, number of other wives, age at first sex, husband educational level, women working status and husband/partners’ age. All these independent variables were retained for North Central and North West. For South East, South South and South West, residing with partner, number of wives, partner’s education was removed with an additional variable, women work status excluded for North East due to collinearity. All analyses were performed using Stata 15.0 at 0.05 level of significance.

#### Generalized linear models

##### Poisson model

The most common technique employed to model count data is Poisson regression. It has a usual feature of equality of mean and variance. Its probability mass function is given as:

1 $${\text{Pr}}\left( {{\text{Y}} = {{\text{y}}_{\text{i}}}{\text{|}}\mu } \right)= \frac{{{{\text{e}}^{{{ - }}\mu }}{\mu ^{{{\text{y}}_{\text{i}}}}}}}{{{{\text{y}}_{\text{i}}}{\text{!}}}};~{{\text{y}}_{\text{i}}}{\text{ = 0}},{\text{1}},{\text{2}}, \ldots$$

Where ${\text{y}}_{\text{i}}$ denote the random variable of the count response, that is, number of children ever born [18, 19].

##### Negative binomial model

The negative binomial (NB) distribution is a two-parameter distribution combining the Poisson distribution and the Gamma distribution (Gamma–Poisson mixture). It relaxes the assumption of equality of mean and variance, thus accounting for unobserved heterogeneity in count data [19–22]. Its probability mass function is given as:

2 $$Pr\left( {{\text{y}}_{\text{i}} {\text{|}} {{\mu }},\alpha } \right) = \frac{{\varGamma \left( {\alpha^{ - 1} + {\text{y}}_{\text{i}} } \right)}}{{\varGamma \left( {\alpha^{ - 1} } \right)\varGamma \left( {{\text{y}}_{\text{i}} + 1} \right) }} \left( {\frac{{\alpha^{ - 1} }}{{\alpha^{ - 1} + {{\mu }}}}} \right)^{{\alpha^{ - 1} }} \times \left( {\frac{{{\mu }}}{{{{\mu }} + \alpha^{ - 1} }}} \right)^{{{\text{y}}_{\text{i}}}} .$$

The mean and variance of the negative binomial distribution are E [y|µ, α] = µ and V [y|µ, α] = µ (1 + αµ). Where α is the dispersion parameter (if α > 0 and µ > 0). Special cases of the negative binomial include the Poisson (α = 0) and the geometric (α = 1) [19].

#### Zero-inflated models

For the zero-inflated Poisson (ZIP), the first process consist of a Poisson distribution that generates counts, some of which may be zero-sampling zero, and the second process is governed by binary distribution (logit or probit) for zero values-structural zeros [23]. Given variable y_i_, The ZIP model probability mass function has two model components as follows:

3 $$\Pr \left( {y_{i} |\mu _{i} } \right) = \left\{ {\begin{array}{*{20}l} {{\text{p}}_{{\text{i}}} + \left( {1 - {\text{p}}_{{\text{i}}} } \right)\exp \left( { - \mu _{{\text{i}}} } \right),} & {{\text{y}}_{{\text{i}}} = 0,0 \le p \le 1} \\ {\frac{{\left( {1 - {\text{p}}} \right)\exp \left( { - \mu _{{\text{i}}} } \right)\mu _{{\text{i}}}^{{{\text{y}}_{{\text{i}}} }} }}{{{\text{y}}_{{\text{i}}} !}}}, & {{\text{y}}_{{\text{i}}} \ge 1} \\ \end{array} } \right.$$

The outcome variable $y_{i}$ is a non-negative integer, $\mu_{i}$ is the expected Poisson count for the ith individual; $p$ is the probability of extra zeros.

Similarly to the ZIP, the zero-inflated negative binomial (ZINB) model is employed to account for both over-dispersion and excess zero problems. For dependent variable y_i_ with many zeros, the ZINB model probability mass function is given as:

4 $$\Pr \left( {y_{i} |\mu _{i} ,\alpha } \right) = \left\{ {\begin{array}{*{20}l} {p_{i} + \left( {1 - p_{i} } \right)\left( {1 + \alpha \mu _{i} } \right)^{{ - \alpha ^{{ - 1}} }} }, & {0 < p < 1} \\ {\left( {1 - p_{i} } \right)\frac{{\Gamma \left( {y_{i} + \frac{1}{\alpha }} \right)\left( {\alpha \mu _{i} } \right)^{{y_{i} }} }}{{y_{{i!}} {\text{ }}\Gamma \left( {\frac{1}{\alpha }} \right)1 + \alpha \mu ^{{y_{i} + \frac{1}{\alpha }}} }}} , & {y_{i} > \alpha } \\ \end{array} } \right.$$

where α ≥ 0 is an over-dispersion parameter [22].

#### Hurdle models

In the hurdle Poisson (HP) model, the first part is the hurdle at zero, which addresses the “few” or “more” zero outcome than the distributional assumption of the Poisson model and the second part governs the truncation part or positive outcomes [2, 19, 23]. Given a variable $y_{i}$. the HP probability distribution is given as:

$$\Pr \left( {y_{i} = 0} \right) = 1 - p, \quad 0 \le p \le 1$$

5 $$\Pr \left( {Y = y_{i} } \right) = p\frac{{\exp \left( { - \mu_{i} } \right)\mu_{i}^{{y_{i} }} }}{{y_{i} !}}, \mu > 0;\quad y_{i} = 1,2, \ldots$$

where µ is the mean of the Poisson model, when $\left( {1 - p} \right) > { \exp }\left( { - \mu } \right)$, the data contain more zeros relative to the Poisson model.

The hurdle negative binomial (HNB) is used when the hurdle model is appropriate and the data exhibit over-dispersion [19, 24]. The HNB model is given as:

$$\Pr \left( {y = 0} \right) = 1 - p, \quad 0 \le p \le 1$$

6 $${ \Pr }\left( {\text{Y = y}} \right) = \frac{\text{p}}{{ 1- \left( {\frac{\text{r}}{{\mu {\text{ + r}}}}} \right)^{\text{r}} }}\frac{{\varGamma ( {\text{y + r)}}}}{{\varGamma \left( {\text{r}} \right){\text{y!}}}}\left[ {\frac{\mu }{{\mu {\text{ + r}}}}} \right]^{\text{y}} \left[ {\frac{\text{r}}{{\mu {\text{ + r}}}}} \right]^{\text{r}} ,\quad {\text{ r,}}\;\mu \;{ > }\; 0 ;\;{\text{y = 1,2}} \ldots$$

The mean and variance of the HNB distribution are given as µ and µ (1 + µ/r) respectively, the quantity µ(1 + µ/r) is a measure of dispersion [22].

#### Model assessment and evaluation

The model selection criterion was based on the maximum likelihood estimates of the model parameter, using the log-likelihood and the Information Criterion (IC)—Akaike (AIC) and Bayesian (BIC). A lower IC value implies that the model is of better fit [25, 26]. An IC values with difference greater than 10 implies that the model with a smaller IC is superior, a value difference of 4 to 10 suggest a moderate superiority of one model against the other and an IC value differences less than 4 implies that the competing models are said to be indistinguishable [26].

## Results

### Socio-economic and demographic characteristics of respondents

**Fig. 1:**
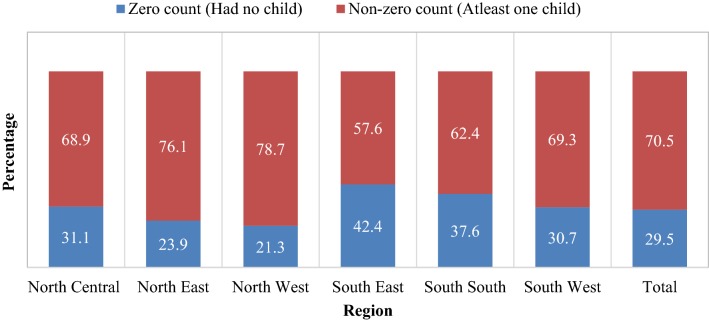
Percentage distribution of zero and non-zero count of children ever born by region (NDHS 2013)

In Nigeria, 29.5% of women age 15 to 49 years had no child, this percentage is highest in South South (42.4) and lowest in North West (21.3) (Fig. 1). The mean number of children ever born was highest in North West (3.89 ± 3.36) and lowest in South South (2.32 ± 2.58). As presented in Table 1, the information reveals that the age at first sex was lower in the Northern part of the country, compared to the Southern part, South East (18.96 ± 4.35), South West (18.69 ± 3.6) and South South (17.27 ± 3.22) except for North Central (18.06 ± 3.78). A higher number of women with no education were recorded in the Northern regions and women wealth quintiles were higher in Southern regions compared to the Northern regions. About 16% of women used any method of contraceptive in Nigeria and this varies across regions.

**Table 1:** Descriptive statistics of background characteristics by region

Region	North Central	North East	North West	South East	South South	South West	All regions
Total (%)	5572 (100)	5766 (100)	11877 (100)	4476 (100)	4942 (100)	6315 (100)	38948 (100)
Residence							
Rural	1521 (27.3)	1579 (27.4)	3402 (28.7)	3149 (70.3)	1913 (38.7)	4850 (76.8)	16414 (42.1)
Urban	4051 (72.7)	4187 (72.6)	8474 (71.3)	1327 (29.7)	3029 (61.3)	1465 (23.2)	22534 (57.9)
Educational level							
None	1763 (31.7)	3711 (64.4)	8240 (69.4)	235 (5.2)	249 (5.0)	531 (8.4)	14729 (37.8)
Primary	1253 (22.5)	791 (13.7)	1382 (11.6)	940 (21.0)	1144 (23.2)	1224 (19.4)	6734 (17.3)
Secondary	2016 (36.1)	980 (17.0)	1956 (16.5)	2668 (59.6)	2845 (57.6)	3463 (54.8)	13927 (35.8)
Higher	540 (9.7)	284 (4.9)	299 (2.5)	634 (14.2)	704 (14.2)	1097 (17.4)	3558 (9.1)
Religion							
Catholic	931 (16.7)	150 (2.6)	314 (2.6)	2114 (47.2)	511 (10.4)	294 (4.6)	4316 (11.1)
Others	2052 (36.8)	775 (13.5)	818 (6.9)	2278 (50.9)	4253 (86.1)	3746 (59.3)	13921 (35.7)
Islam	2434 (43.7)	4788 (83.0)	10605 (89.3)	14 (0.3)	100 (2.0)	2208 (35.0)	20149 (51.7)
Others	155 (2.8)	54 (0.9)	139 (1.2)	71 (1.6)	76 (1.5)	66 (1.1)	561 (1.4)
Ethnicity							
Yoruba	567 (10.2)	29 (0.5)	105 (0.9)	14 (0.3)	120 (2.4)	4646 (73.6)	5482 (14.1)
Hausa/Fulani	404 (7.3)	2315 (40.2)	10288 (86.6)	40 (0.9)	16 (0.3)	201 (3.2)	13263 (34.0)
Igbo	131 (2.4)	18 (0.3)	130 (1.1)	4375 (97.7)	471 (9.5)	512 (8.1)	5636 (14.5)
Others	4470 (80.2)	3404 (59.1)	1354 (11.4)	47 (1.1)	4336 (87.7)	956 (15.1)	14566 (37.4)
Wealth quintiles							
Poorest	567 (10.2)	2224 (38.6)	4036 (34.0)	189 (4.2)	21 (0.4)	95 (1.5)	7132 (18.3)
Poorer	1111 (19.9)	1491 (25.8)	3488 (29.4)	550 (12.3)	419 (8.5)	369 (5.8)	7428 (19.1)
Middle	1726 (31.0)	886 (15.4)	1867 (15.7)	1114 (24.9)	1196 (24.2)	697 (11.1)	7486 (19.2)
Richer	1185 (21.3)	664 (11.5)	1462 (12.3)	1293 (28.9)	1585 (32.1)	1803 (28.5)	7992 (20.5)
Richest	984 (17.6)	501 (8.7)	1024 (8.6)	1330 (29.7)	1721 (34.8)	3350 (53.1)	8910 (22.9)
Contraceptive use							
None	4774 (85.7)	5585 (96.9)	11324 (95.4)	3285 (73.4)	3528 (71.4)	4224 (66.9)	32722 (84.0)
Folkloric	29 (0.5)	9 (0.2)	37 (0.3)	16 (0.4)	69 (1.4)	90 (1.4)	250 (0.6)
Traditional	115 (2.1)	15.7 (0.3)	29 (0.2)	532 (11.9)	396 (8.0)	546 (8.7)	1634 (4.2)
Modern	654 (11.7)	156 (2.7)	486 (4.1)	643 (14.4)	949 (19.2)	1454 (23.0)	4342 (11.2)
Women working status							
No	1708 (30.7)	3098 (53.7)	5012 (42.2)	1589 (35.5)	1671 (33.8)	1605 (25.4)	14685 (37.7)
Yes	3835 (68.8)	2643 (45.8)	6799 (57.3)	2856 (63.8)	3244 (65.7)	4683 (74.2)	24060 (61.8)
Missing	29 (0.5)	25 (0.4)	65 (0.5)	31 (0.7)	26 (0.5)	26 (0.4)	203 (0.5)
Age at first sex							
Never	975 (17.5)	672 (11.7)	1239 (10.4)	936 (20.9)	755 (15.3)	1009 (16.0)	5586 (14.3)
< 18	2192 (39.3)	3601 (62.4)	8551 (72.0)	1276 (28.5)	2007 (40.6)	1925 (30.5)	19552 (50.2)
18+	1930 (34.64)	1183 (20.5)	1610 (13.6)	1970 (44.0)	1884 (38.1)	3114 (49.30	11692 (30.0)
Don’t know	475 (8.5)	310 (5.4)	475 (4.01)	294 (6.6)	296 (6.0)	266 (4.2)	2118 (5.4)
Mean ± SD	18.06 ± 3.78	15.95 ± 3.05	15.09 ± 2.71	18.96 ± 4.35	17.27 ± 3.22	18.69 ± 3.60	16.95 ± 3.66

### Model selection criteria for the fitted model

The model assessments for each of the region are presented in Table 2 using the values from the AIC and BIC for evaluation basis. The hurdle negative binomial model was of best fit for North West (AIC = 45,421.19, BIC = 45,775.64) and South East (AIC = 13,767.37, BIC = 14,026.82) while the zero-inflated negative binomial provided a better fit for North East (AIC = 24,565.28, BIC = 24,828.33). Although, the zero-inflated negative binomial has a moderate superiority over the hurdle negative binomial in South South (AIC = 16,138.5, BIC = 16,411.23). For South West region, both AIC and BIC suggest that ZNB and ZIP are indistinguishable as best fit ($$ZINB \le ZIP < HNB \le HP < NB < Poisson)$$ and no superiority exist between the zero-inflated models and their hurdle model analogs. In all cases, the zero-modified models were better than the GLMs, except for North Central were the BIC suggest that NB is of best fit ($$NB < HNB < ZINB < HP < ZIP < Poisson)$$ contrary to the AIC and the log-likelihood ($$HNB < ZINB < HP < ZIP < NB < Poisson)$$. Similarly, the models which take into account an over-dispersion parameter were better than their corresponding models not accounting for over-dispersion.

**Table 2:** Model assessment for alternative models

Model selection criteria	Fitted models					
	Poisson	NB	ZIP	ZINB	HP	HNB
North Central						
DF	25	26	50	51	50	51
Loglikelihood	− 7421.56	− 7306.18	− 7253.18	− 7224.78	− 7249.53	− 7216.54
AIC	14,893.11	14,664.36	14,606.37	14,551.56	14,599.07	14,535.08
BIC	15,048.30	14,825.76	14,916.75	14,865.13	14,909.45	14,851.67
North East						
DF	19	20	38	39	38	39
Loglikelihood	− 14,780.34	− 12607.10	− 12802.35	− 12243.64	− 12805.15	− 12261.91
AIC	29,598.67	25,254.21	25,680.70	24,565.28	25,686.30	24,601.82
BIC	29,726.82	25,389.10	25,936.99	24,828.33	25,942.60	24,828.33
North West						
DF	25	26	50	48	50	51
Loglikelihood	− 24,795.29	− 22,881.19	− 23,568.25	− 22,679.93	− 23,561.91	− 22,659.60
AIC	49,640.59	45,814.39	47,236.50	45,455.85	47,223.83	45,421.19
BIC	49,814.34	45,995.09	47,583.99	45,789.45	47,571.32	45,775.64
South East						
DF	20	21	40	41	40	41
Loglikelihood	− 8369.57	− 7439.37	− 6887.56	− 6848.18	− 6881.34	− 6842.69
AIC	16,779.13	14,920.73	13,855.13	13,778.35	13,842.68	13,767.37
BIC	16,905.69	15,053.62	14,108.25	14,037.80	14,095.80	14,026.82
South South						
DF	20	21	40	41	40	41
Loglikelihood	− 9129.94	− 8375.15	− 8099.40	− 8028.25	− 8099.58	− 8031.54
AIC	18,299.88	16,792.30	16,278.79	16,138.50	16,279.15	16,145.07
BIC	18,432.92	16,932.00	16,544.88	16,411.23	16,545.23	16417.80
South West						
DF	20	21	40	41	40	41
Loglikelihood	− 11,155.07	− 10,890.47	− 10,162.28	− 10,158.6	− 10,188.28	− 10,187.45
AIC	22350.14	21,822.95	20,404.56	20,399.20	20,456.56	20,456.89
BIC	22,482.74	21,962.18	20,669.75	20,671.03	20,721.76	20,722.39

## Discussion

This study examined the effectiveness of zero-augmented models compared to the standard Poisson and negative binomial models widely used for modelling fertility in Nigeria [3, 9, 11]. The current analysis was conducted separately in each of the six regions in Nigeria.

The results using the AIC and BIC has a model selection reviewed that both hurdle negative binomial and zero-inflated negative binomial provide a better fit for fertility data with large number of zeros and over-dispersion. Extensively, the AIC and BIC estimates from the zero-augmented negative binomial based models (HNB and ZINB) were of better fit than their Poisson based counterparts or in rare cases maybe indistinguishable. Consequently, both excess zeros and over-dispersion were recommended for fertility modelling not only at national level but also at regional levels. These findings are similar to other studies with similar data generating mechanism, containing large number of zeros [24, 27, 28]. Previous studies have noted that zero-inflated models are statistically appropriate in low fertility population studies and especially when there are large number of women with no children [13, 29].

The adjudged best model for each of the regions was used to predict the determinants of fertility peculiar to each region. For North Central, women with at least secondary level of education, partners with secondary education and women not working are factors driving low fertility. Secondary education, Igbo and higher age at first sex are factors determining low fertility in the North East. Residing in rural areas, secondary education, tertiary education, poorer women compared to poor women, no other wives, higher age at first sex and women not working are factors determining low level of fertility in the North West. Urban residence, women not working and increasing women educational level are factors responsible for low level of fertility in the South East. Increasing level of women education, wealth index, high age at first sex and women not working are drivers of low fertility in South South. Secondary and higher level of education, urban residency and women not working are factors contributing to low fertility level in the South West (Additional file 2).

In conclusion, the assessment in this paper provides evidence to support that fertility count data usually rightly skewed with excess zeros should be modelled using the zero-augmented models with negative binomial variant.

## Limitation

Children ever born (CEB) was captured in NDHS based on the reported full birth history of women of reproductive age. There is likelihood of gross under-reporting of CEB due to cultural beliefs and norms of reporting actual number of births.

## Supplementary information

**Additional file 1.** Summary statistics of children ever born by region.

**Additional file 2.** Determinant of fertility by regions based on the adjudged best model.

## Data Availability

This study used a secondary dataset from Measure DHS program, the dataset can be accessed after due permission from the DHS program archive and can be downloaded at https://dhsprogram.com/data/dataset/Nigeria_Standard-DHS_2013.cfm?flag=0.

## Abbreviations

AIC: Akaike Information Correction
BIC: Bayesian Information Correction
CEB: children ever born
DF: degree of freedom
EAs: enumeration areas
HNB: hurdle negative binomial
HP: hurdle Poisson
IC: information criterion
NB: negative binomial
SD: standard deviation
TFR: total fertility rate
ZIP: zero-inflated Poisson
ZINB: zero-inflated negative binomial
