# Supplementary material for: Robustness of zero-augmented models over generalized linear models in analysing fertility data in Nigeria
Source: BMC Res Notes. 2019 Dec 18;12:815. doi: 10.1186/s13104-019-4852-5 (PMC6921497; doi:10.1186/s13104-019-4852-5)
Supplement: Supplementary file 1 — Additional file 1. Summary statistics of children ever born by region. [file 13104_2019_4852_MOESM1_ESM.docx]

| Summary Statistics of Children Ever Born by Region | | | | | |  |  |
| --- | --- | --- | --- | --- | --- | --- | --- |
| CEB | N^f^ | Min | Max | Mean | SD | Skewness | Pr(Skewness) |
| North Central | 6251 | 0 | 13 | 2.72 | 2.64 | 0.79 | 0.000 |
| North East | 6630 | 0 | 17 | 3.52 | 3.24 | 0.72 | 0.000 |
| North West | 9673 | 0 | 17 | 3.89 | 3.36 | 0.60 | 0.000 |
| South East | 4462 | 0 | 12 | 2.45 | 2.91 | 0.97 | 0.000 |
| South South | 6058 | 0 | 18 | 2.32 | 2.58 | 1.00 | 0.000 |
| South West | 5874 | 0 | 14 | 2.42 | 2.31 | 0.81 | 0.000 |
| Total | 38948 | 0 | 18 | 3.07 | 3.03 | 0.87 | 0.000 |

^f^Unweighted frequencies
