# Supplementary material for: Robustness of zero-augmented models over generalized linear models in analysing fertility data in Nigeria
Source: BMC Res Notes. 2019 Dec 18;12:815. doi: 10.1186/s13104-019-4852-5 (PMC6921497; doi:10.1186/s13104-019-4852-5)
Supplement: Supplementary file 2 — Additional file 2. Determinant of fertility by regions based on the adjudged best model. [file 13104_2019_4852_MOESM2_ESM.docx]

| Determinant of Fertility by Regions Based on the Adjudged Best Model. | | | | | | | | | |
| --- | --- | --- | --- | --- | --- | --- | --- | --- | --- |
| Regions | North East | South West | South South | North Central | North West | | South East | |  |
|  | ZINB | | | HNB | | | | |  |
| Variables | AIRR (95% CI) | AIRR (95% CI) | AIRR (95% CI) | AIRR (95% CI) | | AIRR (95% CI) | | AIRR (95% CI) |  |
| ***Residence*** |  |  |  |  | |  | |  |  |
| Rural | 0.93 (0.78-1.10) | 1.05 (0.98-1.13) | 0.96 (0.88-1.04) | 1.01 (0.94-1.09) | | 0.87* (0.79-0.94) | | 1.05 (0.97-1.13) |  |
| ***Educational level*** |  |  |  |  | |  | |  |  |
| Primary | 0.96 (0.87-1.06) | 0.93 (0.86-1.01) | 0.91* (0.85-0.98) | 0.94 (0.86-1.03) | | 0.97 (0.90-1.04) | | 0.79* (0.74-0.85) |  |
| Secondary | 0.65* (0.59-0.72) | 0.71* (0.66-0.77) | 0.56* (0.51-0.60) | 0.66* (0.61-0.73) | | 0.74* (0.67-0.93) | | 0.45* (0.39-0.51) |  |
| Higher | 0.74* (0.63-0.86) | 0.53* (0.48-0.58) | 0.53* (0.47-0.60) | 0.63* (0.55-0.72) | | 0.79* (0.67-0.93) | | 0.47* (0.41-0.54) |  |
| ***Religion*** |  |  |  |  | |  | |  |  |
| Other Christians | 1.16* (1.05-1.27) | 1.06 (0.95-1.18) | 1.04 (0.92-1.18) | 0.97 (0.87-1.07) | | 0.84 (0.69-1.02) | | ### |  |
| Islam | 1.04 (0.92-1.17) | 0.99 (0.89-1.11) | 0.98 (0.79-1.22) | 0.87* (0.79-0.97) | | 0.93 (0.74-1.16) | | ### |  |
| Others | 1.34 (0.98-1.84) | 0.94 (0.78-1.14) | 1.09 (0.91-1.31) | 0.94 (0.81-1.08) | | 1.01 (0.79-1.30) | | ### |  |
| ***Ethnicity*** |  |  |  |  | |  | |  |  |
| Hausa/Fulani | 0.73 (0.53-1.01) | 1.31* (1.18-1.46) | 1.08 (0.55-2.12) | 0.92 (0.78-1.07) | | 1.28* (1.06-1.53) | | ### |  |
| Igbo | 0.54* (0.36-0.83) | 1.10 (1.00-1.22) | 0.78* (0.63-0.96) | 0.98 (0.76-1.26) | | 1.04 (0.81-1.34) | | ### |  |
| Others | 0.68* | 0.88* (0.81-0.95) | 0.83* (0.70-0.98) | 1.03 (0.94-1.12) | | 1.24* (1.03-1.49) | | ### |  |
| ***Wealth Quintiles*** |  |  |  |  | |  | |  |  |
| Poorer | 0.99 (0.91-1.07) | 1.15* (1.05-1.27) | 0.73* (0.57-0.94) | 0.97 (0.84-1.11) | | 0.93* (0.89-0.98) | | 1.02 (0.97-1.07) |  |
| Middle | 0.95 (0.86-1.05) | 1.18* (1.06-1.32) | 0.75* (0.60-0.95) | 1.02 (0.90-1.16) | | 0.95* (0.88-1.02) | | 0.53* (0.28-0.97) |  |
| Richer | 1 | 1.1 (0.98-1.24) | 0.78* (0.62-0.98) | 0.96 (0.83-1.10) | | 0.99 (0.90-1.09) | | 1.07 (0.96-1.20) |  |
| Richest | 1.06 (0.86-1.30) | 1.02 (0.90-1.15) | 0.72* (1.03-1.20) | 0.96 (0.82-1.12) | | 0.93 (0.80-1.07) | | 1.09 (0.98-1.22) |  |
| ***Contraceptive use*** |  |  |  |  | |  | |  |  |
| Folkoloric | 1.43* (1.06-1.93) | 1.09 (0.91-1.32) | 1.17* (1.00-1.37) | 1.49* (1.20-1.85) | | 0.84 (0.70-1.01) | | 1.13 (0.76-1.69) |  |
| Traditional | 1.18 (0.9-1.54) | 1.22* (1.15-1.30) | 1.08 (0.98-1.18) | 1.16* (1.01-1.34) | | 1.14 (0.93-1.38) | | 1.27* (1.19-1.37) |  |
| Modern | 1.23* (1.1-1.38) | 1.20* (1.14-1.25) | 1.12* (1.03-1.20) | 1.22* (1.13-1.32) | | 1.16* (1.03-1.32) | | 1.33* (1.21-1.47) |  |
| ***Residing with partner*** |  |  |  |  | |  | |  |  |
| No | ### | ### | ### | 0.90 (0.82-1.00) | | 0.91 (0.81-1.04) | | ### |  |
| ***Partner have other wives*** |  |  |  |  | |  | |  |  |
| Yes | ### | ### | ### | 1.13* (1.07-1.19) | | 1.13* (1.08-1.17) | | ### |  |
| ***Age at first sex*** |  |  |  |  | |  | |  |  |
| 18+ | 0.79* (0.74-0.85) | 0.84* (0.80-0.87) | 0.88* (0.84-0.93) | 0.85* (0.80-0.90) | | 0.81* (0.75-0.88) | | 0.87* (0.82-0.92) |  |
| ***Partner's education*** |  |  |  |  | |  | |  |  |
| Primary | ### | ### | ### | 1.02 (0.93-1.13) | | 0.86* (0.82-0.91) | | ### |  |
| Secondary | ### | ### | ### | 0.88* (0.80-0.96) | | 0.79* (0.74-0.85) | | ### |  |
| Higher | ### | ### | ### | 1.08 (0.99-1.19) | | 0.97 (0.88-1.06) | | ### |  |
| ***Women working status*** |  |  |  |  | |  | |  |  |
| Yes | ### | 1.55* (1.39-1.73) | 1.69* (1.55-1.85) | 1.29* (1.21-1.36) | | 1.26* (1.19-1.33) | | 1.63* (1.47-1.80) |  |
| constant | 6.71* (4.59-9.83) | 2.59* (2.12-3.15) | 5.01* (3.62-6.92) | 3.85* (3.09-4.80) | | 4.16* (3.23-5.37) | | 5.49 (3.91-7.70) |  |
| alpha | 0.29* (0.25-0.88) | 0.02* (0.01-0.08) | 0.14* (0.10-0.18) | 0.07* (0.05-0.11) | | 0.22* (0.19-0.24) | | 0.09* (0.06-0.12) |  |

**P-value significant at 0.05; AIRR-Adjusted Incidence Relative Ratio; ### -expunged due to collinearity; Reference category: Residence (Urban), Educational Level (None), Religion (Catholic), Ethnicity (Yoruba), , Wealth index (Poor), Contraceptive Use (None), Residing with partner (No), Age at first sex (< 18), Partner's education level (None), women working status (No).*
